# Newly described human polyomaviruses Merkel Cell, KI and WU are present in urban sewage and may represent potential environmental contaminants

**DOI:** 10.1186/1743-422X-7-141

**Published:** 2010-06-28

**Authors:** Sílvia Bofill-Mas, Jesus Rodriguez-Manzano, Byron Calgua, Anna Carratala, Rosina Girones

**Affiliations:** 1 Department of Microbiology, Faculty of Biology, Universitat de Barcelona, Av. Diagonal 645, 08028 Barcelona, Spain

## Abstract

Recently, three new polyomaviruses (KI, WU and Merkel cell polyomavirus) have been reported to infect humans. It has also been suggested that lymphotropic polyomavirus, a virus of simian origin, infects humans. KI and WU polyomaviruses have been detected mainly in specimens from the respiratory tract while Merkel cell polyomavirus has been described in a very high percentage of Merkel cell carcinomas. The distribution, excretion level and transmission routes of these viruses remain unknown.

Here we analyzed the presence and characteristics of newly described human polyomaviruses in urban sewage and river water in order to assess the excretion level and the potential role of water as a route of transmission of these viruses. Nested-PCR assays were designed for the sensitive detection of the viruses studied and the amplicons obtained were confirmed by sequencing analysis. The viruses were concentrated following a methodology previously developed for the detection of JC and BK human polyomaviruses in environmental samples. JC polyomavirus and human adenoviruses were used as markers of human contamination in the samples. Merkel cell polyomavirus was detected in 7/8 urban sewage samples collected and in 2/7 river water samples. Also one urine sample from a pregnant woman, out of 4 samples analyzed, was positive for this virus. KI and WU polyomaviruses were identified in 1/8 and 2/8 sewage samples respectively. The viral strains detected were highly homologous with other strains reported from several other geographical areas. Lymphotropic polyomavirus was not detected in any of the 13 sewage neither in 9 biosolid/sludge samples analyzed.

This is the first description of a virus isolated from sewage and river water with a strong association with cancer. Our data indicate that the Merkel cell polyomavirus is prevalent in the population and that it may be disseminated through the fecal/urine contamination of water. The procedure developed may constitute a useful tool for studying the excreted strains, prevalence and transmission of these recently described polyomaviruses.

## Findings

Human polyomaviruses JC and BK (JCPyV and BKPyV) are two members of the *Polyomaviridae *family that persistently infect humans and cause disease in immunocompromised individuals. These viruses have been potentially implicated in certain cancers [1,2]. Both respiratory and oral routes have been postulated for their transmission [3-5]. A high frequency of excretion of JCPyV and BKPyV has been reported, and both viruses have been detected in urban sewage from various geographical areas [6,7]. This observation indicates that they could be transmitted by water or food.

In 2007 and 2008, three new polyomaviruses, KI WU and Merkel cell polyomavirus (KIPyV, WUPyV and MCPyV), were reported in humans [8-10]. KIPyV and WUPyV have been detected mainly in respiratory tract specimens from children and also immunocompromised individuals. In 4 continents these viruses showed equivalent prevalence and highly conserved nucleotide sequences. KIPyV and WUPyV have also been co-detected with other viruses in patients with respiratory and, in some cases, gastrointestinal disorders. Both viruses have been detected in feces [11,12] and their role in the etiology of respiratory infections has recently been questioned [13].

MCPyV, which has also been described in respiratory secretions [14-16], is strongly associated with Merkel cell carcinomas (MCC) [17]. This association strongly supports an etiological role for MCPyV in the development of MCC [18]. Recent serological data show that KIPyV, WUPyV and MCPyV are prevalent in the healthy population [19].

Antibodies against lymphotropic polyomavirus (LPyV), a virus of simian origin, have been found in human blood samples [19,20]. Moreover, LPyV has been reported in human peripheral blood from patients with leukoencephalopathies as well as in immunocompromised and healthy subjects [21,22].

Here we assessed KIPyV, WUPyV, MCPyV and LPyV in urban wastewater to determine whether these viruses are prevalent in the environment, as reported for JCPyV and BKPyV [7]. For this purpose, we performed nested-PCR (nPCR) assays and compared our results with the nucleotide sequences available in data banks. Wastewater samples collected over the last 6 years from a treatment plant processing domestic and industrial wastewater from a population of 175,000 inhabitants were tested for the presence of KIPyV, WUPyV and MCPyV (8 sewage samples) and also for LPyV (13 sewage and 9 biosolid and sludge samples). In addition, 7 samples collected in 2009 from river water used to source a drinking water treatment plant were also analyzed for the presence of KIPyV, WUPyV and MCPyV. The presence of JCPyV and human adenoviruses (HAdVs) was evaluated by quantitative PCR (qPCR) as a control of the procedures applied and as an index of the level of fecal pollution of human origin present in the samples [6].

Urine samples collected from 4 healthy pregnant women were also tested for WUPyV, KIPyV and MCPyV.

Viral particles were concentrated using methods developed in a previous study using JCPyV as a model. Metods were based on: ultracentrifugation and elution of samples with glycine buffer pH 9.5 for sewage [7] and sludge or biosolids [6], glass wool columns filtration and glycine buffer elution for river water [23] and on ultracentrifugation for urine [3]. Negative controls were established for each batch of samples. Nucleic acids were extracted with the QIAamp Viral RNA kit (QIAGEN, Inc.). Oligonucleotide primers (Table 1) were designed based on existing polyomaviral sequences and their specificity against other known polyomaviruses (JCPyV, BKPyV, SV40, LPyV) was checked by nPCR. Samples were analyzed by nPCR in final 50-μL reaction volumes. Briefly, 10 μL of the extracted nucleic acids (corresponding to 2 mL of sewage, 2.5 mL of sludge, 1 g of biosolids, 13.5 mL of river water, and 2 mL of urine) and a 10-fold dilution (to prevent enzymatic inhibition) of each nucleic acid extraction were analyzed in a 40-μL reaction mixture containing 1xPCR Buffer, MgCl_2 _at 1.5 mM, 0.025 mM of each dNTP, 0.5 μM of primers and 2 units of TaqGold DNA polymerase (Applied Biosystems). After a first-round PCR, 1 μL of the product was added to 49 μL of the nPCR mixture containing the same components as the first-round PCR mixture. The conditions for the first-round and nPCR reaction conditions were as follows: 95°C for 10 min, 30 cycles of 94°C for 60 sec, 60 sec at the corresponding annealing temperature (Table 1) and extension at 72°C for 60 sec. Amplification was completed with a 7-min extension step at 72°C. Amplicons of the expected size were purified (QIAquick PCR purification kit, QIAGEN, Inc) and sequenced (BigDye sequencing kit and ABI Prism 377 genetic analyzer; Applied Biosystems).

**Table 1:** Oligonucleotide primers used for nPCR amplification of WUPyV, KIPyV, MCPyV and simian polyomavirus LPyV

Primer	Virus region	Position	Amplification reaction	Product size (bp)	Annealing temperature (°C)	Sequence (5'-3')
WU1	WUPyV (VP1)^a^	1730-1750	First	505	55	CCCACAAGAGTGCAAAGCCTTC
WU2		2234-2213				AGGCACAGTACCATTGGTTTTA
WU3		2044-2063	Nested	164	50	AGTTTTGGTGCTTCCTKTSC
WU4		2207-2188				TACAGTATACTGAGCAGGC

KI1	KIPyV (VP1)^b^	1684-1704	First	378	59	GCTGCTCAGGATGGGCGTGA
KI2		2061-2043				CAGKGTTCTAGGGTCTCCTGGT
KI3		1899-1918	Nested	190	54	GTTGCTTGTTGTACCTCTAG
KI4		2088-2067				AATTGTATAGGTAGTTGGGCCT

MC1	MCPyV (TAg)^c^	1716-1736	First	477	55	GCCTGTGAATTAGGATGTATTT
MC2		2210-2198				CATTTCTGTCCTGGTCATTTCCA
MC3		2010-2033	Nested	183	50	GCCCATTATCTAGACTTTGCAAA
MC4		2192-2173				TCTAACCTCCTTTTGGCTA

MC1b	MCPyV (VP1)^c^	3174-3194	First	440	58	GGCTTTCTTTTTGAGAGGCCT
MC2b		3613-3592				AGTGGGCCCTCTATGCAAAGGA
MC3b		3276-3297	Nested	240	54	TTGGGTAAACAGTTTTCTCCTG
MC4b		3515-3493				TGCCTAGATATTTTAATGTTACT

MC1c	MCPyV (VP1/2/3)^c^	4228-4252	First	265	53	GAATTAACTCCCATTCTTGGATTCA
MC2c		4492-4472				TTGGCTTCTTCCTCTGGTACT
MC3c		4264-4286	Nested	198	53	ATTTGGGTAATGCTATCTTCTCC
MC4c		4461-4439				GGATATATTTCTCCTGAATTACA

LN1	LPyV(VP2/VP3)^d^	1542-1564	First	423	54	GGCACACCAAAGAGTAACTCAAG
LN2		1965-1943				CAGGTCATGTCTTCATTTAGGAG
LN3		1617-1639	Nested	232	54	GGAAGTGGAGCTTAATAAATTGG
LN4		1863-1849				ATATCCATACAAGTCCTCAGAAG

VP1, VP2 and VP3 = Virion protein 1, 2 and 3; TAg = T antigen; K= G +T; S = G + C

^a ^The sequence positions are referred to strain EF444549

^b ^The sequence positions are referred to strain EF127906

^c ^The sequence positions are referred to strain EU375803

^d ^The sequence positions are referred to strain K02562

Nucleotide sequences were analyzed using the basic BLAST program http://www.ncbi.nlm.nih.gov/BLAST/. Separate areas were used for the diverse steps of the procedures developed; non-template controls were included in each nPCR reaction. HAdV and JCPyV were tested as a control of the procedures applied as well as of the presence of enzymatic inhibitors in the samples.

We processed the samples as 3 separate batches at 3 separate periods of time. The samples showed typical levels of human fecal pollution, as shown by JCPyV and HAdV concentrations (Table 2). KIPyV and WUPyV were present in 1/8 and 2/8 sewage samples respectively while MCPyV was present in 7/8 sewage samples and was the unique newly described human polyomavirus found in the river water (Table 2). MCPyV was also detected in 1/4 urine samples. The VP1 and VP1/VP2/VP3 genes of the MCPyV genome were also amplified and sequenced in 3 sewage samples to confirm the presence of MCPyV genome (Table 2).

**Table 2:** Presence of human polyomaviruses and human adenoviruses in sewage and river water samples

Samples, type	Collection date (month/year)	Quantitative PCR (GC/mL of sample)		Nested-PCR results (presence/absence)		
		
		HAdV	JCPyV	WUPyV	KIPyV	MCPyV (Tag)
BCN1, sewage	02/2004	2.81 × 10^3^	1.35 × 10^3^	+	-	+^a^
BCN2, sewage	07/2007	4.29 × 10^3^	7.94 × 10^2^	-	-	+^a^
BCN3, sewage	07/2007	1.57 × 10^3^	NT	-	-	+^a^
BCN4, sewage	07/2007	6.10 × 10^3^	8.65 × 10^2^	-	-	+^a, b^
BCN5, sewage	05/2008	Non tested	5.48 × 10^2^	+^a^	+^a^	+^b^
BCN6, sewage	09/2006	9.40 × 10^1^	7.65 × 10^2^	-	-	+
BCN7, sewage	11/2006	1.35 × 10^2^	4.83 × 10^2^	-	-	+^b^
BCN8, sewage	12/2006	6.00 × 10^2^	8.33 × 10^1^	-	-	-
BCN9, river water	03/2009	3.08 × 10^0^	1.00 × 10^0^	-	-	-
BCN10, river water	03/2009	7.90 × 10^0^	9.40 × 10^0^	-	-	+^a^
BCN11, river water	03/2009	1.10 × 10^1^	1.21 × 10^1^	-	-	+^a^
BCN12, river water	03/2009	1.18 × 10^1^	1.49 × 10^1^	-	-	-
BCN13, river water	03/2009	1.99 × 10^0^	4.40 × 10^0^	-	-	-
BCN14, river water	03/2009	2.48 × 10^0^	1.21 × 10^1^	-	-	-
BCN15, river water	03/2009	3.46 × 10^0^	9.94 × 10^0^	-	-	-

NT = Not tested

^a ^Sequenced amplicons

^b ^Samples from other regions (VP1 and/or VP1/VP2/VP3) in which MCPyV has been amplified and sequenced (GQ452776, GQ390249-50)

Although the detection technique used here was not quantitative, limiting-dilution nPCR experiments showed approximately 10-100 PCR units/mL of sewage for KIPyV, WUPyV and MCPyV. Samples showed positive results only after nPCR but not after the first-round PCR.

DNA cross contamination was ruled out since no viral strains or plasmids with the genomes of the viruses were available, only for LPyV was a plasmid available in the laboratory as positive control; however, all samples were found to be negative for this virus.

We found that the viruses showed a high degree of sequence stability. All but one sequenced MCPyV amplicon were identical and also identical to the reference sequence with GenBank accession number: EU375803, despite their distinct origins (sewage, river water or urine). This observation confirms the high level of conservation of the DNA of these viruses. Only one MCPyV VP1 amplicon showed a nucleotide that differed from the others and from strain EU375803 although it does not produce any change in the derived protein sequence.

The WUPyV amplicon sequenced was identical to reference strain EF444549 while the KIPyV amplicon sequenced showed one nucleotide of difference with reference strain EF127906.

The nucleotide sequences obtained were deposited in GenBank [GenBank: GQ376529 (WUPyV), GQ376528 (KIPyV), GQ376530 (MCPyV TAg region), GQ452776 (MCPyV VP1/VP2-VP3 region) and GQ390249/50 (MCPyV VP1 region)].

None of the 22 sewage, sludge and biosolid samples tested positive for LPyV although typical concentrations of JCPyV and HAdV indicated human fecal contamination (data not shown). The nPCR assay showed a sensitivity of 1-10 genomic copies/reaction when the complete LPyV genome [24] cloned in pBR322 and quantified spectrophotometrically was analyzed by limiting-dilution nPCR. Thus, LPyV was not detected in the tested samples by these methods.

The observation that MCPyV DNA was much more frequently detected than that of KIPyV or WUPyV might reflect that MCPyV is a more prevalent infection or that it is a highly excreted virus.

Our results on MCPyV in urine, urban sewage and river water strongly support the notion that this virus shows an excretion pattern that resembles that of JCPyV and BKPyV. Human excretion of new polyomaviruses, especially MCPyV, may lead to fecal (urine) contamination of water and food.

In this study we did not attempt the *in vitro *culture of the new polyomaviruses because no cell culture systems for these viruses are available at present. Furthermore, for other human polyomaviruses, such as JCPyV, the regulatory regions of strains excreted in urine present an archetypal structure and are inefficiently cultured.

To our knowledge, this is the first report of the presence of a virus strongly related to human cancer in sewage and river water samples. We propose that the methodology reported here is suitable to study the prevalence, excretion pattern and genetic variability of recently discovered human polyomaviruses in environmental matrices.

## Competing interests

The authors declare that they have no competing interests.

## Authors' contributions

SBM coordinated the study, concentrated urine samples and nucleic acid extractions of the urine samples, collaborated in PCR assays, typed the amplicons detected and drafted the manuscript. JRM concentrated the sewage and biosolid samples and performed the nucleic acid extractions; he also collaborated in the PCR analysis and in the sequencing of the resulting amplicons. BC concentrated river water samples and performed nucleic acid extraction of the same samples. AC collaborated in the production of standards for the quantification of HAdV and JCPyV and in the nucleotide sequence comparisons. RG participated in the development of the methodology, conception and coordination of the study and helped to draft the manuscript. All authors read and approved the final manuscript.

## Authors' information

SBM is an assistant professor at the Department of Microbiology of the Faculty of Biology, University of Barcelona. Her main research interests are the epidemiology of human and animal polyomaviruses. She addresses their transmission through the environment and their potential as indicators of the presence of human or/and animal fecal contamination.
